# Zoledronic Acid Supplementation With Excision of a Giant Cell Tumor of the Distal End Radius to Prevent Recurrence: A Case Report

**DOI:** 10.7759/cureus.55794

**Published:** 2024-03-08

**Authors:** Ashutosh Lohiya, Nareshkumar Dhaniwala, Shivshankar Jadhav, Siddharth K Patel, Saksham Goyal

**Affiliations:** 1 Orthopaedics, Jawaharlal Nehru Medical College, Datta Meghe Institute of Higher Education and Research, Wardha, IND

**Keywords:** bisphosphonates, intralesional curettage, zoledronic acid, giant cell tumor, distal end radius

## Abstract

Giant cell tumors are benign yet locally aggressive neoplasms commonly observed in the distal radius, exhibiting higher recurrence rates compared to other tumor types. This study presents a case involving a 50-year-old farmer who presented with swelling at the distal end of his wrist. The patient underwent treatment involving intralesional curettage and supplementation with zoledronic acid, resulting in a significant reduction in the tumor's potential for recurrence. This approach aims to achieve an optimal balance between functional outcomes and disease management in the majority of cases. While this strategy proves effective in most instances, there may be scenarios where resection becomes imperative due to the severity of the disease, ensuring adequate disease clearance. In such circumstances, judicious decision-making coupled with an appropriate treatment plan is crucial to guarantee a satisfactory outcome, even in the face of challenges.

## Introduction

The giant cell tumor (GCT), a benign yet locally aggressive bone tumor, is characterized by sheets of ovoid, neoplastic mononuclear cells interspersed with large cells that resemble osteoclasts [1]. Representing around 20% of benign bone lesions and 4-5% of malignant bone cancers, GCTs are most prevalent in individuals aged 20 to 45, constituting 70% of cases, while occurrences in children under ten are rare. GCTs typically affect the ends of long bones, particularly the distal radius, a critical site for radiocarpal articulation and hand functionality. The removal of distal radius tumors poses challenges in repairing the resulting damage [1]. Recent attention has focused on surgery for GCT combined with zoledronic acid supplementation, a bisphosphonate serving as an adjuvant treatment to prevent recurrence. Zoledronic acid induces apoptosis in osteoclasts, precursors of GCT of the bone (GCTB), contributing to recurrence prevention. However, managing GCTs at the lower end of the radius is complex due to intricate anatomy, the need for a high disease clearance rate, and the imperative for optimal functional outcomes [2]. While the axial skeleton, particularly the sacrum, is commonly affected by GCT, the appendicular skeleton sees higher prevalence in the lower end of the femur, the upper end of the tibia, the lower end of the radius, and the upper end of the humerus. The lower end of the radius stands out as the third most frequently affected site [3].

## Case presentation

**Figure 1:**
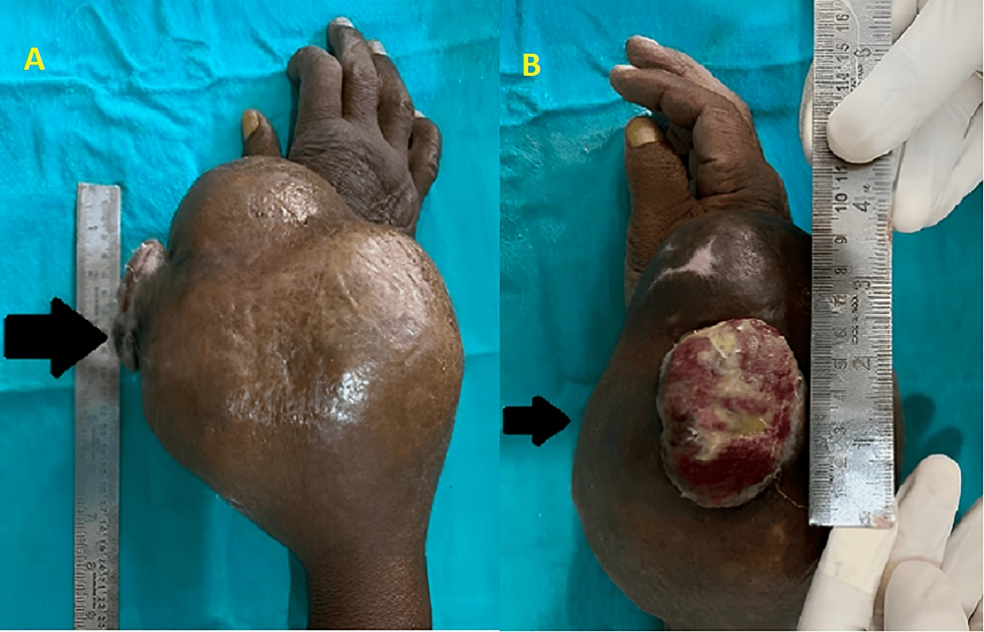
Large heterogeneously enhancing expansile multilocular lesion with a wound over the lateral aspect of the distal end of the radius - dorsal plane (A) and lateral plane (B)

A 50-year-old farmer arrived at the hospital's emergency department, reporting swelling and pain in his right wrist that had persisted for the past year. Additionally, he mentioned a discharge from the swelling site over the last 30 days. He recounted a fall on his outstretched hand two years ago, leading to an X-ray at a government hospital. The radiograph revealed an abnormality, and surgical intervention was recommended. However, the patient opted for conservative management, including medication and plaster application, which successfully reduced the swelling. Further history disclosed another trauma to the same right wrist a year ago, resulting in insidious swelling and gradually increasing pain. Due to residing in a rural area and apprehensions about surgery, the patient refrained from seeking additional treatment. After six months, there was a sudden surge in swelling and intensified pain, impeding daily activities and eventually causing a complete loss of functionality. In the last 30 days, he also observed pus discharge from the swelling site, exhibiting a yellowish to greenish color, thick mucoid consistency, and a foul odor. The swelling progressed into a cauliflower-like outgrowth, with a wound on the radial aspect rupturing and enlarging over the past three to four weeks. Upon examination, a circumferential growth was evident on the right wrist, along with a fungating cauliflower-like mass on the radial aspect. The lobulated swelling displayed multiple lobes on the right wrist's medial, lateral, and dorsal aspects, as illustrated in Figure 1. The patient is unable to do daily activities like holding a pen, combing hair, etc. The patient also complained of a loss of sensation in the distal part of the limb.

**Figure 2:**
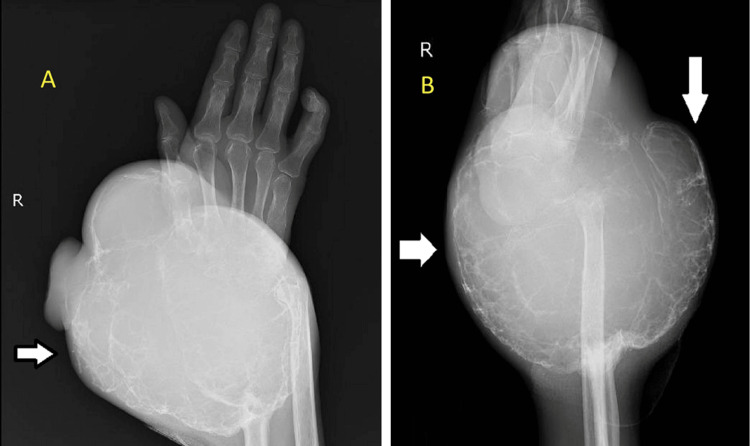
X-ray of the right wrist in anteroposterior (A) and lateral (B) views showing soap bubble appearance

The patient underwent an X-ray of the right wrist as depicted in Figure 2, which is suggestive of a large expansile multilocular lesion at the distal end of the radius with a soap bubble appearance. As the patient was not able to sense anything distally from the swelling and the swelling was progressing rapidly, an accelerated method of management was required to prevent its further spread. The potential clinical diagnoses considered were telangiectatic osteosarcoma or malignant transformation of a GCT with a secondary aneurysmal bone cyst component.

**Figure 3:**
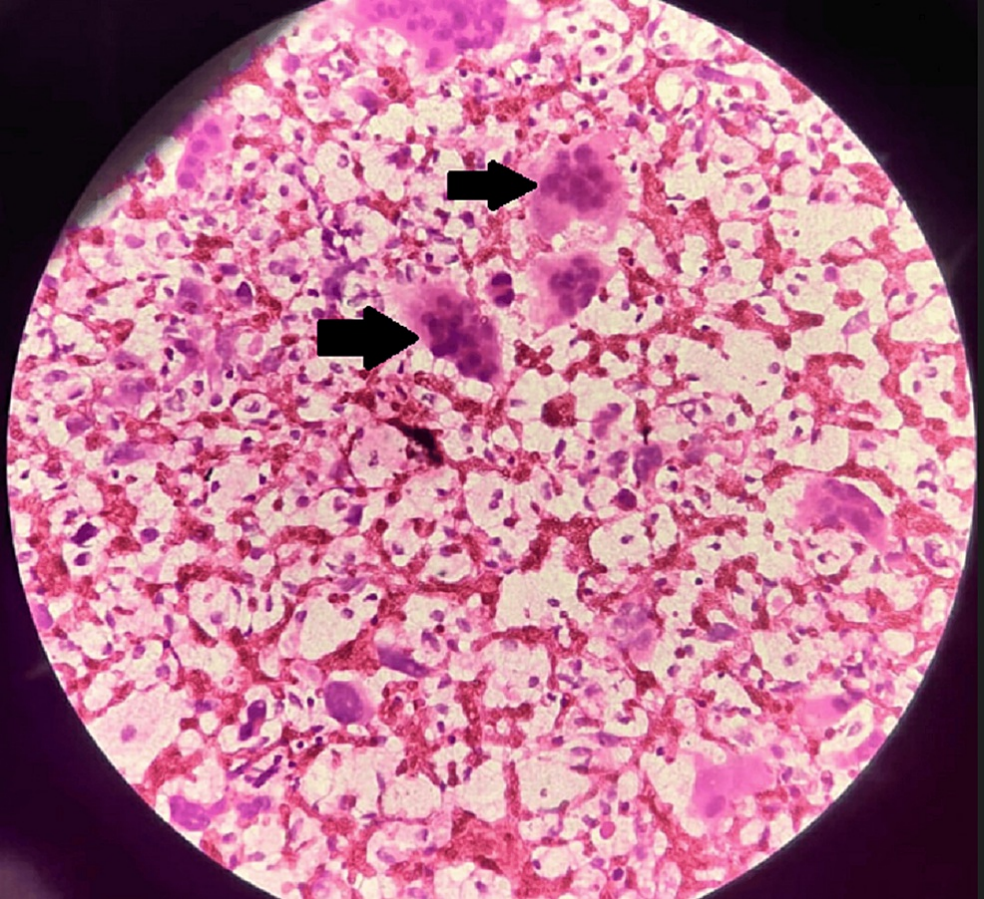
Histopathology (40x) of the right wrist showing giant cells as depicted by arrows

**Figure 4:**
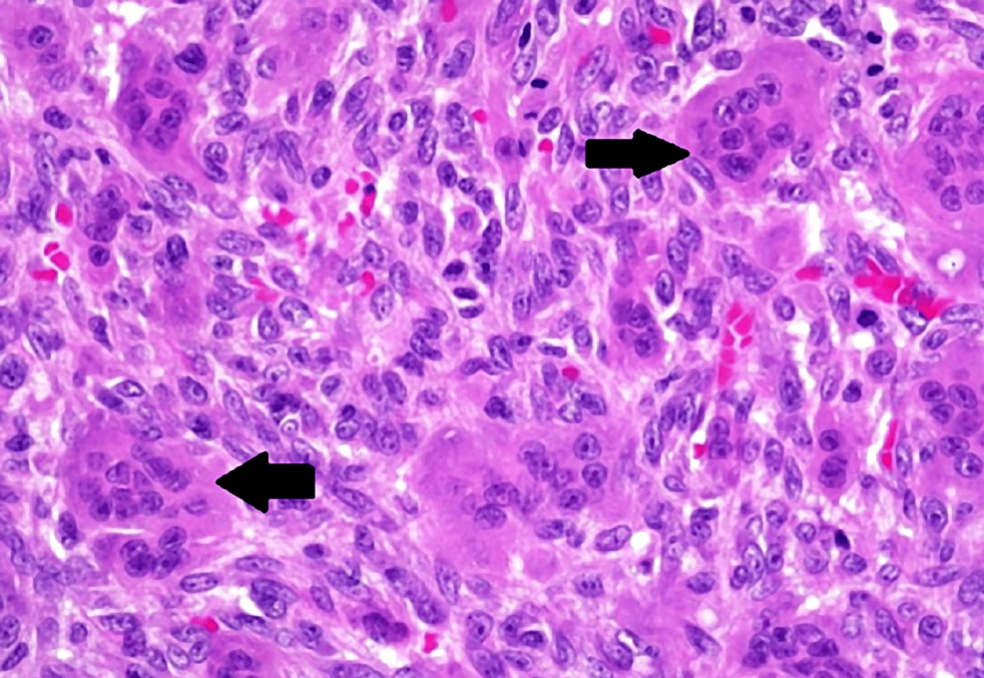
Histopathology (100x) of the right wrist showing giant cells as depicted by arrows

Given the large tumor and complete loss of functionality, surgery was planned. Routine labs were done as a part of the pre-op evaluation. The patient was screened for metastasis, but there were no palpable lymph nodes over the mediastinum and paraaortic areas, and the patient had no other symptoms in the chest. A multidisciplinary team decided on a limb-sparing strategy that included broad resection with amputation below the elbow and adjuvant therapy with zoledronic acid because of the degree of bone involvement and the risk of recurrence connected with traditional surgical excision. The patient underwent the surgery under spinal anesthesia and a tourniquet application. He underwent below-elbow amputation, and stump closure was achieved. Tissue from the amputated hand was sent for histopathology to confirm the diagnosis. The histopathological examination of the sample in Figures 3-4 revealed the presence of giant cells in the given section.

To reduce the chance of tumor recurrence, the patient's treatment regimen included adjuvant therapy with zoledronic acid. Zoledronic acid was used to stop osteoclastic activity and reduce the likelihood of recurrence. Zoledronic acid treatment has been linked to a lower rate of tumor recurrence in patients with GCTBs who have undergone surgery. Thus, it is advised to administer zoledronic acid after a thorough and intensive tumor curettage procedure.

## Discussion

Young adults are more frequently affected by GCT compared to their older counterparts. Approximately 50% of all tumors occur in individuals in their third and fourth decades of life [4]. GCTs account for four to ten percent of primary bone tumors and 15% to 20% of benign bone tumors, with a rare occurrence in patients over the age of fifty. The female-to-male ratio in GCTs ranges from 1.3 to 1.5 to 1, and the incidence is notably higher in Asian populations compared to Western populations [4]. The knee joint is the most common site for these tumors, representing 44% of cases, followed by the distal radius (10%), proximal humerus (6%), and hands/feet (13%) [4]. The spine and skull are rarely affected, with the ala of the sacrum being the most frequently impacted site in the axial skeleton. The vertebral body is the most commonly affected location in the spine, while the distal extremities are a typical site [4].

Despite their benign nature, GCTs can exhibit aggressive growth patterns and local spread. Metastasis occurs in 1-5% of cases, and there is a correlation between metastasis, local aggressiveness, and recurrence. The most common site for metastasis is the lungs. Local aggressiveness can result in significant local effects, such as cortical breakthrough or spread into nearby articular structures and soft tissues, with a recurrence rate of approximately 35%. Before the age of 20, GCTs were sporadic, accounting for less than 5% of all cases, with skeletally immature patients more likely to have vertebral GCT and multicentricity [4-5]. While less common, multifocal lesions tend to be more aggressive than isolated lesions. Patients with Paget's disease are more prone to GCT, with the skull and pelvis being the most commonly affected flat bones. The lung is where GCTs of bone most frequently metastasize; reports of this type of tumor range from 1% to 9%. Its occurrence in many places, including the breast, bone, skin, and lymph nodes (mediastinum and paraaortic), has been documented in isolated case reports. However, more people are aware of these tumors' ability to spread, and better detection techniques have made them more noticeable.

Radiographically, GCT exhibits a unique radiolucent geographic appearance with a short transition zone at the lesion boundary. CT scans are more effective in assessing bone mineralization, cortical thinning, and penetration than conventional radiography. MRI is valuable for evaluating soft tissue integrity, subchondral extension into neighboring joints, and neurovascular systems. A typical GCT appears well-circumscribed on MRI with homogenous signal intensity, low on T1-weighted images, and intermediate on T2-weighted images [6]. The limb salvage option for this case was considered, such as intralesional curettage with autograft reconstruction and, if needed, arthrodesis of the wrist joint, but the size of the tumor was progressing rapidly and there was extensive local spread of the tumor cells along with a wound over the lateral aspect of the wrist. The case was further discussed with the onco-surgeon, and the patient was given both options: limb salvage and amputation. The patient wanted a one-time intervention and opted for amputation at the below elbow site. Surgical resection was selected, which is the gold standard for treating GCT, with some authors recommending an intralesional technique to preserve bone architecture over excision, especially in young adults where most GCTs are benign and located near joints.

Zoledronic acid, a bisphosphonate, has gained popularity as an adjuvant therapy for GCTBs [7]. It induces apoptosis in GCTB precursor osteoclasts. Zoledronic acid-loaded cement has shown cytotoxicity to GCT cell lines and can reduce recurrence after intralesional curettage [8]. It can also be utilized to prevent tumor recurrence [9-10]. Telangiectatic osteosarcoma and malignant transformation of GCTs with a secondary aneurysmal bone cyst component are considered differential diagnoses [9]. IV zoledronic acid is a helpful adjuvant to surgery in GCT treatment because it reduces pain, causes sclerosis, and triggers apoptosis. These effects reduce the rate at which tumors advance and the rate at which local bone is destroyed [11]. In surgically treated GCTB patients, zoledronic acid treatment lowers the incidence of tumor recurrence. As a result, using zoledronic acid after aggressive, prolonged tumor curettage is recommended. More carefully thought-out randomized controlled trials will support this data.

## Conclusions

Given that GCTs are the most prevalent benign bone tumor and can display aggressiveness with a notable recurrence rate, early detection and prompt treatment become crucial. The lower end of the radius is the third most frequently affected site. In rural settings, where access to specialized care may be limited, prioritizing early detection and intervention is essential to prevent further complications. To enhance outcomes in the adjuvant therapy of GCT with zoledronic acid and to advance the early detection of these tumors, it is imperative to promote strategies that improve care, coordination, and communication. This may involve implementing protocols for regular screenings, facilitating communication between healthcare providers, and fostering a collaborative approach to patient care. By emphasizing these measures, healthcare professionals can contribute to more effective management and better outcomes for individuals with GCTs, particularly in settings where resources may be constrained.
